# Supplementary material for: Micronuclei Detection by Flow Cytometry as a High-Throughput Approach for the Genotoxicity Testing of Nanomaterials
Source: Nanomaterials (Basel). 2019 Nov 24;9(12):1677. doi: 10.3390/nano9121677 (PMC6956333; doi:10.3390/nano9121677)
Supplement: Supplementary file 1 [file nanomaterials-09-01677-s001.pdf]

Supplementary Material

Micronuclei Detection by Flow Cytometry as a High-Throughput Approach for the Genotoxicity Testing of Nanomaterials

Alba García-Rodríguez [1], Liliya Kazantseva [1], Laura Vila [1], Laura Rubio [1], Antonia Velázquez [1,2], María José Ramírez [3], Ricard Marcos [1,2,*] and Alba Hernández [1,2,*]

[1] Department of Genetics and Microbiology, Faculty of Biosciences, Universitat Autònoma de Barcelona, 08193 Bellaterra, Spain; albagr.garcia@gmail.com (A.G.-R.); liliyak1292@gmail.com (L.K.); lauravilavecilla@hotmail.com (L.V.); laurarubio33@hotmail.com (L.R.); Antonia.Velazquez@uab.cat (A.V.)

[2] Consortium for Biomedical Research in Epidemiology and Public Health (CIBERESP), Carlos III Institute of Health, 28029 Madrid, Spain

[3] Consortium for Biomedical Research on Rare Diseases (CIBERER), Carlos III Institute of Health, 28029 Madrid, Spain; MariaJose.Ramirez@uab.cat

* Correspondence: ricard.marcos@uab.es (R.M.); alba.hernandez@uab.es (A.H.); Tel.: +34-93-581-20-52 (R.M); Fax: +34-93-581-23-87 (R.M. and A.H.)

PROTOCOL FOR THE FCMN ASSAY

1. After cell exposure in 6-well plates, the medium is removed (by aspiration). Cells are washed twice with 1X PBS, and collected by trypsinization (1x trypsin in 1X PBS). To inactivate the trypsin, culture medium is added. The resuspension is done for each individual well. Cells are collected in individual tubes (FACS tubes), and centrifuged at 500 g for 8 min. After centrifugation, the supernatant is aspirated leaving about 50 µL. The cells are resuspended by mild agitation.

2. To each tube 125 µL of specific nucleic acid staining [0.125 mg/mL EMA (ethidium monozide bromide) (25 µL) prepared in 1X PBS with 2% inactivated bovine fetal serum (100 µL)] is added. The samples are dipped at a depth of 2 cm in ice. A light source (60 W lamp) is applied about 30 cm above the tubes for about 20 min. Then, it is added to each sample 900 µL of PBS 1X cold, with 2% of inactivated bovine fetal serum, protecting the tubes with foil. Tubes are centrifuged at 500 g for 8 min and. after centrifugation the supernatant is aspirated, leaving about 50 µL.

3. The cells are resuspended by mild agitation and, to each tube, 125 µL of a solution of lysis 1 (0.584 mg/mL of NaCl, 1 mg/mL of sodium citrate, 0.3 µL/mL IGEPAL, 1 mg/mL RNasa A and 0.2 µM of SYTOX Green prepared in deionized water) is added, slowly. The tubes are briefly stirred with the vortex (medium speed) and incubated at room temperature for 1 h. Finally,125 µL of a solution of lysis 2 (85.6 mg/mL of sucrose, 15 mg/mL of citric acid and 0.2 µM of SYTOX Green, prepared in deionized water, are added. Samples are kept at room temperature for 30 min before the flow cytometry assay.

FCMN analysis

In our study, we used a Cytometer FACSCalibur (Becton Dickinson) with the following description: Cytometer equipped with an argon laser (emission at 488 nm) and another of red diode (emission at 635 nm). This device allows working with six parameters (FCS, SSC and 4 fluorescence) as standard optical configuration.

Figures 1 and 2 show the different steps carried out to debug the obtained results. Only data from a negative (Figure1) and positive (Figure 2) controls are indicated, as a model. As observed, the original FC data (A,A') correspond to the obtained cells/nuclei, once debris has been eliminated. Next step is "doublet discrimination" where "double cells" are eliminated (B,B'). Two further steps eliminating dots according size (C,C') and complexity (D,D') are done. Finally, those EMA positive dead cells (E,E') are eliminated. After all these steps, dots are classified in three windows corresponding to nuclei, hypodiploid nuclei, and micronuclei.

The values of the FC analysis can be tabulated as indicated in Table 1

Table S1. Examples of positive and negative control data obtained in duplicated.

Treatment		Nuclei	MN	Hypodiploid	MN/1000	Mean MN/1000
Positive control	MMC A	9,681	1,454	77	150.19	
	MMC B	9,731	1,525	104	156.72	
	(A + B)	19,412	2,979	181		153.45
Negative control	C- A	9,811	133	34	13.56	
	C- B	9,856	126	30	12.78	
	(A + B)	19,667	239	64		14.13

FIGURE 1. NEGATIVE CONTROL

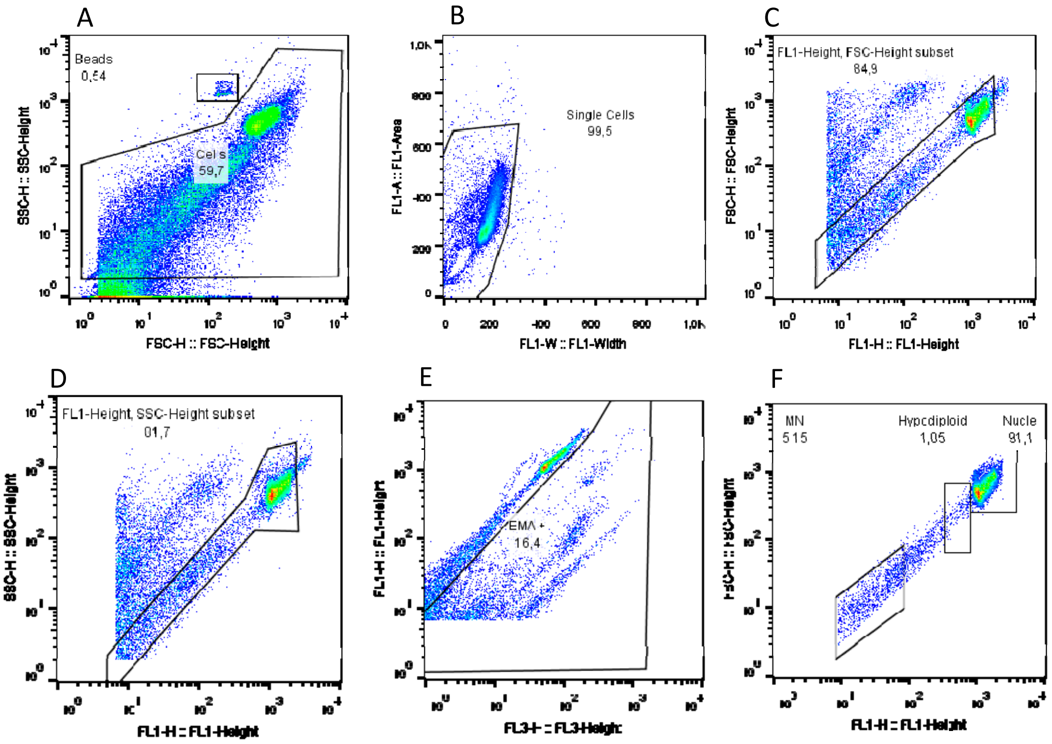

Figure S1. Negative control

FIGURE 2. POSITIVE CONTROL

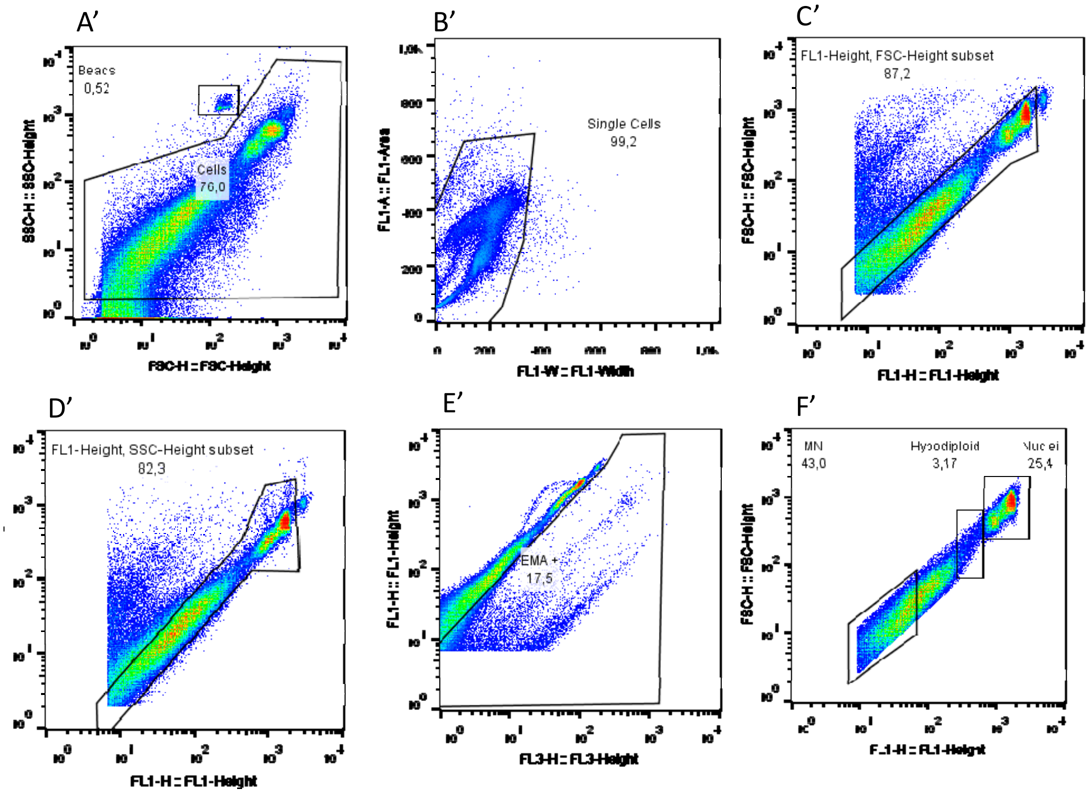

Figure S2. Positive control
